# Eye Drop with Fas-Blocking Peptide Attenuates Age-Related Macular Degeneration

**DOI:** 10.3390/cells13060548

**Published:** 2024-03-20

**Authors:** Yujong Yi, Seon-Hong Pyun, Chae-Yeon Kim, Gyeongju Yun, Eunhwa Kang, Seoyoun Heo, Irfan Ullah, Sang-Kyung Lee

**Affiliations:** 1 Department of Bioengineering and Institute of Nanoscience and Technology, Hanyang University, Seoul 04763, Republic of Korea; yiyujong@gmail.com (Y.Y.); seoyoun1217@gmail.com (S.H.); 2 Department of Internal Medicine, Yale University, New Haven, CT 06520, USA

**Keywords:** age-related macular degeneration (AMD), eye drops, Fas-blocking peptide, necrosis, dry AMD, wet AMD, apoptosis, vascular endothelial growth factor (VEGF) inhibitors, anti-VEGF agents, retina

## Abstract

Age-related macular degeneration (AMD), characterized by macular retinal degeneration, poses a significant health concern due to the lack of effective treatments for prevalent dry AMD. The progression of AMD is closely linked to reactive oxygen species and Fas signaling, emphasizing the need for targeted interventions. In this study, we utilized a NaIO_3_-induced retinal degeneration mouse model to assess the efficacy of Fas-blocking peptide (FBP). Intravitreal administration of FBP successfully suppressed Fas-mediated inflammation and apoptosis, effectively arresting AMD progression in mice. We developed a 6R-conjugated FBP (6R-FBP) for eye drop administration. 6R-FBP, administered as an eye drop, reached the retinal region, attenuating degeneration by modulating the expression of inflammatory cytokines and blocking Fas-mediated apoptosis in rodent and rabbit NaIO_3_-induced retinal degeneration models to address practical concerns. Intravitreal FBP and 6R-FBP eye drops effectively reduced retinal degeneration and improved retinal thickness in rodent and rabbit models. This study highlights the therapeutic potential of FBP, particularly 6R-FBP as an eye drop, in inhibiting Fas-mediated cell signaling and protecting against retinal cell death and inflammation in dry AMD. Future investigations should explore the translational prospects of this approach in primates with eye structures comparable to those of humans.

## 1. Introduction

Age-related macular degeneration (AMD) is a prominent cause of vision impairment among many individuals aged over 50 years [1]. This condition specifically damages the macula, a crucial component of the retina that is responsible for clear and central vision [2]. AMD is broadly classified into two categories: dry AMD (nonexudative, atrophic) and wet AMD (exudative, neovascular) [3,4]. Dry AMD accounts for approximately 90% of AMD cases, presenting distinct challenges compared to wet AMD [5,6,7]. Although wet AMD can be treated with FDA-approved intravitreal anti-vascular endothelial growth factor (VEGF) drug injections, such as Lucentis and Eylea, these treatments are not universally responsive, are invasive, and are associated with potential side effects [5,6,7,8]. To address the challenges of intravitreal injection, alternative drug delivery routes, such as topical administration, have been investigated for AMD [9,10,11]. Unfortunately, ongoing research has yet to yield a viable treatment option for dry AMD, prompting the use of symptomatic approaches involving antioxidants and vitamins [5,12].

The pathogenesis of dry AMD involves persistent mild inflammation during the early stages, coupled with the accumulation of drusen deposits in the subretinal space, which eventually leads to irreversible loss of the photoreceptor and retinal pigment epithelium (RPE) in the macula [5]. As dry AMD progresses, vision loss intensifies, culminating in geographic atrophy (GA), with approximately 10% of patients transitioning to wet AMD [5,13]. Necrosis and apoptosis within the macula region of the retina may result from oxidative damage in the retina and choroid, driven by an imbalance between local oxidants and antioxidant systems, leading to chronic inflammation [14,15]. Despite our understanding of these processes, the precise mechanisms underlying AMD pathogenesis are not fully understood [15].

Fas receptor (Fas, CD95)-mediated apoptosis in retinal cells is a pivotal contributor to AMD [16,17]. In the early stages of AMD, the nuclear factor kappa-light-chain-enhancer of activated B cells (NF-κB) pathways are activated through the interaction of Fas with the Fas ligand (FasL) and upon damage induced by intracellular reactive oxygen species (ROS) [18,19,20]. This activation triggers an increase in inflammation and cytokine levels within the retina [21,22]. Proinflammatory cytokines, particularly TNF-α and IFN-γ, stimulate Fas upregulation through an NF-κB-dependent autocrine loop [20,23,24]. Additionally, ROS-induced p53 upregulates Fas expression, sensitizing retinal cells to apoptosis [25,26,27,28,29]. As the inflammatory response progresses, FasL-expressing macrophages infiltrate the retinal layers, interacting with Fas-expressing RPE cells, thereby triggering Fas-mediated apoptosis [30,31]. Upon activation of Fas signaling, apoptosis is initiated through both the intrinsic and extrinsic pathways [32,33]. Activated caspase-8, resulting from Fas signaling, accelerates the intrinsic pathway, promoting mitochondrial ROS generation through truncated BID (tBID) [34,35]. Moreover, Fas-signaling not only activates the c-Jun N-terminal kinase (JNK) and NF-κB pathways, it also triggers the hyperactivation of extracellular signal-regulated kinases (ERK) pathways through intracellular ROS generation [21,36,37,38,39]. This dual activation contributes significantly to the intrinsic pathway of apoptosis in the retina, and the interplay of these signaling cascades amplifies the inflammatory response and reinforces the apoptotic process.

Given this information, we hypothesized that Fas-blocking peptide (FBP) could be a novel therapeutic target for AMD treatment. Previous studies have demonstrated that FBP binding to Fas inhibits ERK signaling and activates NF-κB signaling [40]. The ERK pathway, particularly under increased ROS conditions, affects the mitochondria and triggers apoptosis [38,41,42,43]. NF-κB signaling plays a crucial role in regulating inflammation and cell survival, inhibiting apoptosis by antagonizing the ROS/JNK signaling pathway [44,45,46]. During apoptosis, caspase-3 cleaves NF-κB through its protease activity, resulting in a decrease in the intracellular levels of NF-κB [47]. Therefore, we hypothesized that the application of FBP to apoptotic cells may stimulate NF-κB activation, blocking the caspase cascade and ERK activation in the Fas-signaling pathway, thereby reducing NF-κB activation via caspase-3 and ERK signaling.

The anatomical and physiological characteristics of static and dynamic ocular barriers pose the primary challenge in ocular delivery systems. Static barriers encompass the precorneal tear film, corneal epithelium, sclera, and the blood–aqueous barrier, which presents an obstacle to effective drug penetration [48,49]. Dynamic ocular barriers involve tear drainage, clearance through conjunctival blood and lymphatic systems, and circulation in choroidal blood and lymphatics, adding layers of complexity to achieving optimal drug distribution [49,50]. Although there are various routes of drug administration, including the use of microcannulas, microcatheters, microneedles, intravitreal injection, and subconjunctival injection, these approaches are often invasive and necessitate repeated injections [49,51]. Accordingly, less invasive topical administration methods, including biodegradable contact lenses and topical eye drops, have been formulated [51]. Noninvasive and efficient ocular drug delivery methods, such as eye drops and cell-penetrating peptides (CPPs), are also being explored as alternative formulation strategies. These strategies address challenges posed by the size of therapeutic molecules, including proteins and peptides [9].

**Figure A1:**
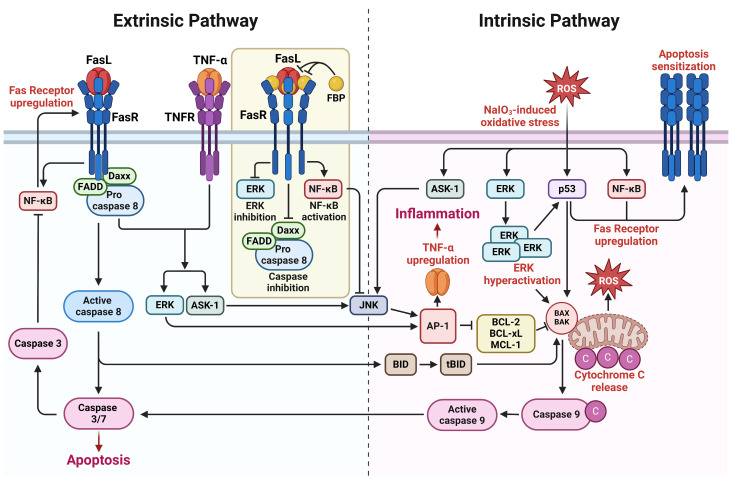
Schematic diagram illustrating the effects of the Fas-blocking peptide (FBP) on extrinsic and intrinsic Fas-signaling pathways. The arrows indicate the direction of influence, providing a visual representation of the regulatory effects exerted by FBP on Fas-mediated signaling. Briefly, the ROS signal in the eyes activates the ERK, p53, and NF-κB pathways. p53 and NF-κB, in turn, induce the upregulation of Fas, pushing the cell into an apoptosis-sensitized state, ultimately leading to the extrinsic apoptosis pathway. The hyperactivation of p53 and ERK, induced by oxidative stress, leads to the transcription of intrinsic apoptosis factors, such as BAX and BAK, initiating the intrinsic apoptosis pathway. FBP binding to Fas disrupts caspase-8-activated apoptosis, resulting in the blocking of the formation of tBID, which is involved in the intrinsic apoptosis pathway and ROS generation. Reduced caspase-8 and caspase-1 levels from ROS signaling downregulate the production of inflammatory cytokines from proinflammatory cytokines.

In this study, we created a novel CPP, 6R-FBP, by conjugating hexa-arginine (6R) with FBP for efficient delivery of the therapeutic peptide to the retina via eye drops. Our data demonstrate that administering 6R-FBP via eye drops effectively delivers FBP to the retinal area in a mouse NaIO_3_-induced retinal degeneration model. Both 6R-FBP and FBP eye drops could inhibit retinal degeneration in a rabbit NaIO_3_-induced retinal degeneration model. These observations suggest that the efficiency of ocular delivery of peptides to retinal cells via eye drops may be influenced by the peptide size, a factor that appears to vary across different species. Our findings indicate a successful attenuation of retinal degeneration in the NaIO_3_-induced retinal degeneration model via the suppression of Fas-signaling, as depicted schematically in Figure A1.

## 2. Materials and Methods

### 2.1. Peptides

The peptides used in this study were synthesized by Anygen Co. (Gwangju, Jeolla-do, Korea). The peptides, received in lyophilized form, were reconstituted in phosphate-buffered saline (PBS, pH 7.4) and stored at −70 °C for experimental purposes. The sequences of the synthesized peptides are depicted in Table 1.

### 2.2. Retinal Pigment Epithelial Cell Culture

The human RPE cell line (ARPE-19) was obtained from ATCC (Rockville, MD) and cultured in Dulbecco’s modified Eagle medium/Nutrient Mixture F-12 (DMEM/F-12), supplemented with 10% fetal bovine serum and 1% penicillin (100 IU/mL). All experiments were performed using cells within specified passages. To mimic the in vitro environment prevalent in AMD, ARPE-19 cells were cultured with 10 mM sodium iodate (NaIO_3_) for 24 h.

### 2.3. Cell Viability Assay

Cell viability was assessed using the CCK-8 assay (Dojindo Laboratories, Kumamoto, Japan). For evaluating the effect of FBP on Fas signaling, ARPE-19 cells were seeded in a 12-well plate (2 × 10^5^ cells/mL), and varying concentrations of FBP (100, 300, 500, 700, and 900 μM) were directly dissolved in media containing NaIO_3_ (10 mM) for treatment. The CCK-8 assay was performed 24 h after treatment, following the manufacturer’s protocol. Absorbance was measured at 450 nm using an INFINITE 200 PRO microplate reader (TECAN, Zurich, Switzerland).

To confirm the cytotoxicity of CPP-conjugated FBP (4R-FBP, 5R-FBP, 6R-FBP, 9R-FBP) on ARPE-19 cells, the cells were plated in a 12-well plate at a density of 2 × 10^5^/mL. After 24 h of treatment with various peptides (4R-FBP, 5R-FBP, 6R-FBP, 9R-FBP, and FBP), the CCK-8 assay was performed as per the manufacturer’s protocol.

### 2.4. In Vitro Real-Time PCR Analysis

To examine the expression levels of the Fas receptor, inflammatory cytokines, and chemokines in ARPE-19 cells, the cells were seeded in a 12-well plate at a density of 2 × 10^5^ cells/mL. After 24 h of treatment with NaIO_3_ (10 mM) and the FBP peptide (700 μM), the cells were harvested for analysis. Total RNA from the ARPE-19 cells was extracted using the RNAiso kit (Takara, Kyoto, Japan). Total RNA was reverse-transcribed into cDNA using the High-Capacity cDNA Reverse Transcription Kit (Applied Biosystems, Waltham, MA, USA). The quantification of gene expression was performed using PowerUp™ SYBR™ Green Master Mix (Thermo Fisher Scientific, Waltham, MA, USA) on an ABI 7500 Fast Real-Time PCR system (Applied Biosystems, Waltham, MA, USA). The sequences of primer pairs used for real-time PCR are depicted in Table 2.

### 2.5. In Vitro Western Blot Analysis

Total protein samples were extracted from ARPE-19 cells using ice-cold RIPA buffer containing a protease inhibitor cocktail. A 4× sample buffer containing Tris-(2-Carboxyethyl)phosphine (Invitrogen, Carlsbad, CA, USA) was prepared by mixing 950 µL of 4× Laemmli sample buffer (GenDEPOT) with 50 µL of Tris-(2-Carboxyethyl)phosphine (Invitrogen). Protein samples (15 μg) were diluted in the sample buffer and heated at 95 °C for 5 min. The samples were then loaded onto a 12% SDS-PAGE gel and transferred onto a PVDF transfer membrane. The blots were probed with rabbit anti-cleaved caspase-3 (1:1000, Abcam, Cambridge, UK, ab52293), anti-cleaved caspase-8 (1:1000, Abcam, ab25901) and rabbit polyclonal anti-Fas (1:1000, Abcam, ab82419). Secondary polyclonal antibodies were probed with rabbit IgG coupled to HRP (1:2000, Abcam, ab97051). Finally, the blots were developed using ECL Western blotting substrate (Promega, Madison, WI, USA).

### 2.6. In Vitro Apoptosis Analysis

The efficacy of FBP on the inhibition of ARPE-19 cell apoptosis was assessed through annexin V/propidium iodide (PI) staining, followed by flow cytometry analysis. ARPE-19 cells were seeded in a 12-well plate (2 × 10^5^ cells/mL). After 24 h of treatment with NaIO_3_ (10 mM) and the FBP peptide (700 μM), the cells were harvested and stained using the Dead Cell Apoptosis Kits with annexin V for Flow Cytometry (Invitrogen, Carlsbad, CA, USA), following the manufacturer’s protocol. The stained cells were collected and analyzed using a BD FACSCalibur™ system (Becton Dickinson, Franklin Lakes, NJ, USA) [52].

### 2.7. Animal Studies

All experiments were conducted in compliance with the guidelines and protocols approved by the Institutional Animal Care and Use Committee (IACUC) of Hanyang University. Retinal degeneration was induced by intravenous injection of NaIO_3_ (30 mg/kg) in 6-week-old male BALB/c mice (*N* = 3 per group), weighing 18–22 g (Orient Bio, Seoul, Korea). The eye drop administration group received peptides dissolved in PBS at dosages of 10 mg/mL (50 μg/5 μL) per ocular instillation. For intravitreal administration, peptide solutions dissolved in PBS were administered into the vitreous humor at a concentration of 1 mg/mL (1 μg/1 μL) per administration.

### 2.8. In Vivo Delivery of Peptides

To confirm the delivery of FBP to the retina, we conducted ex vivo biodistribution imaging (FOBI; CELLGENTEK, Deajeon, Korea) and immunohistochemistry (IHC) to verify the delivery of Alexa Fluor™ 647 (Alexa647)-conjugated peptides. The N-terminal of the target peptide was fluorescently tagged using Alexa Fluor™ 647 NHS Ester (Succinimidyl Ester) (Invitrogen, Carlsbad, CA, USA), following the manufacturer’s protocol. Subsequently, free dye molecules were removed through a PD-10 desalting column (GE Healthcare, Milwaukee, WI, USA). Seven days after induction with NaIO_3_, Alexa647-conjugated peptides were administered via eye drops. Twelve hours after the last administration, organs were harvested for experiments, and Alexa647 signals were detected on whole organs using a FOBI imaging machine (CELLGENTEK, Cheongju, Republic of Korea).

For IHC, eyes were harvested and embedded in the Tissue-Tek^®^ OCT Compound (Sakura Finetek Europe BV, Alphen aan den Rijn, The Netherlands) to prepare cryoblocks for cryosectioning. After freezing the eyes in the Tissue-Tek^®^ OCT Compound, the eyes were cryosectioned to a thickness of 10 μm and mounted with ProLong™ Glass Antifade Mountant with NucBlue™ Stain (Invitrogen). The anti-Fas rabbit mAb (#4233S, Cell Signaling Technology, Danvers, MA, USA) was used to capture the Fas receptor, and the Fas antibody was subsequently detected using Goat Anti-Rabbit IgG H&L (Alexa Fluor^®^ 488) (ab150077, Abcam, Cambridge, UK). A ZEISS Axio Scan.Z1 Slide Scanner (Carl Zeiss, Oberkochen, Germany) was employed to detect NucBlue™ stained cell nuclei and the Alexa647-conjugated peptide signals.

### 2.9. In Vivo Real-Time PCR Analysis

The mouse eye posterior hemisphere was dissected using Vannas scissors. Subsequently, total RNA was extracted from the posterior hemisphere of the left eye using the RNAiso kit (Takara). RNA was reverse-transcribed with the High-Capacity cDNA Reverse Transcription Kit (Applied Biosystems, Waltham, MA, USA) and quantified using PowerUp™ SYBR™ Green Master Mix (Thermo Fisher Scientific, Waltham, MA, USA) on an ABI 7500 Fast Real-Time PCR system (Applied Biosystems, Waltham, MA, USA). Relative expression was determined using the ΔΔCt method, normalized against *GAPDH* mRNA levels. The primer sequences used are depicted in Table 2.

### 2.10. H&E Staining and TUNEL Assay

To confirm the therapeutic effect of 6R-FBP, histological evaluation was conducted through hematoxylin and eosin (H&E) staining and the TUNEL assay [53]. The eyes were fixed with 4% paraformaldehyde in PBS at room temperature for 20 min. After fixation, the eyes were sequentially incubated in 10%, 20%, and 30% (*w*/*v*) sucrose solution diluted in PBS for 30 min each at room temperature. Subsequently, the eyeballs were sagittally halved with a razor blade and embedded in Tissue-Tek^®^ OCT Compound, and then frozen immediately in a liquid nitrogen chamber. Trimming was minimized to maintain controlled distances and ensure consistency, and sections were sliced at a thickness of 10 μm. Sections from the eye cryoblock were used for H&E staining and the TUNEL assay. The TUNEL assay was conducted according to the manufacturer’s protocol using the TUNEL assay kit (Abcam).

### 2.11. NaIO_3_-Induced Retinal Degeneration Rabbit Model and FBP Administration

The efficacy of the treatment was evaluated through an experiment conducted by KNOTUS, a contract research organization. In brief, retinal degeneration was induced by intravenous injection of NaIO_3_ (60 mg/kg) in 20 chinchilla rabbits, weighing 2 kg.

The eye drop administration group received dosages of 5 mg/mL (250 μg/50 μL) per ocular instillation. For intravitreal administration, peptide solutions (5 mg/mL) were administered in increments of 40 μL per administration. A pipette was used to administer the test substance onto the central cornea of the right eye for each experimental group.

Anesthetized animals were administered the test substance via subconjunctival injection into the right eye using a 31-gauge syringe needle attached to a syringe. In the case of eye drop administration, the peptides and PBS were administered twice daily for 2 weeks, for a total of 28 administrations. For intravitreal administration, a single dose of the peptide was administered the day after the NaIO_3_ injection.

### 2.12. Fundus Imaging in NaIO_3_-Induced Retinal Degeneration Rabbit Model

Before the administration of NaIO_3_ (Day 0) and upon concluding the experiment (Day 14), the right eyes of the rabbits were treated with a mydriatic agent (1% midriacyl eye drops). Subsequently, the rabbits were anesthetized with a mixture of Zoletil^®^50 (Virbac, Carros, France) and Rompun^TM^ (Bayer AG, Leverkusen, Germany) through intravenous injection (marginal ear vein) at a dosage of 0.1 mL/kg. Fundus imaging was conducted using a fundus camera (TRC-50IX, TOPCON, Tokyo, Japan). The retinal images were analyzed using Image J software version 1.54d (NIH, Bethesda, MD, USA) to determine the area of degeneration in the RPE, with the RPE injury area being manually selected. The total area of the retina, excluding the optic nerve region, was defined as the total area. The proportion of degenerated area was calculated as a percentage using Equation (1).

Degenerated area (%) = Degenerated area/Total area(1)

### 2.13. OCT Imaging in NaIO_3_-Induced Retinal Degeneration Rabbits

Before concluding the experiment (Day 14), optical coherence tomography (OCT) imaging was conducted on the right eye of all animals. The imaging was centered on two sections, Upper and Lower, relative to the optic nerve. Thereafter, the retinal thickness was analyzed at three points in each image. The average of these values was calculated, resulting in a total of six values.

### 2.14. H&E Staining in NaIO_3_-Induced Retinal Degeneration Rabbits

On the concluding day of the experiment (Day 14), the animals were anesthetized using the previously described mixture of Zoletil^®^50 and Rompun^TM^, after which they were sacrificed. NaIO_3_-induced retinal degeneration eyes were then extracted, and the central cornea was punctured using an injection needle. Subsequently, the specimens were fixed in a 10% neutral buffered formalin solution. For histopathological examination, the fixed tissues were subjected to processes such as fixation, dehydration, paraffin embedding, and sectioning. H&E staining was then performed, and the proportion (%) of the thickness of the outer nuclear layer (ONL) to the overall retinal thickness was calculated using an Olympus BX-53 optical microscope (Olympus, Tokyo, Japan). Two slides were prepared per eyeball relative to the optic nerve. Measurements were taken at three points on each slide, amounting to a total of six values, which were averaged.

### 2.15. Statistical Analysis

For all experiments, statistical comparisons among groups were conducted using appropriate methods. To assess statistical differences in mean values between groups, we utilized either the Mann–Whitney *U* test or one-way ANOVA. The Graphpad Prism 10 software was used for statistical analysis versus mock group. A *p*-value *<* 0.05 was considered statistically significant.

## 3. Results

### 3.1. FBP Reduces Inflammation and Apoptosis in NaIO_3_-Induced Oxidative Stress in ARPE-19 Cells

In a previous study, we successfully demonstrated the effective inhibition of Fas/FasL ligation by FBP that disrupted Fas-signaling in neuronal cells [54,55]. To evaluate the safety profile of FBP, we assessed its cytotoxic effects on a human retinal pigment epithelial cell line (ARPE-19). FBP exhibited no cytotoxicity at concentrations up to 900 μM (Figure S1). Subsequently, we investigated the protective effects of FBP against NaIO_3_-induced oxidative stress. The viability of NaIO_3_-induced ARPE-19 cells was maximum at FBP concentrations exceeding 700 μM (Figure S2) [56]. Therefore, we maintained a consistent FBP concentration of 700 μM throughout the experiments.

**Figure 1:**
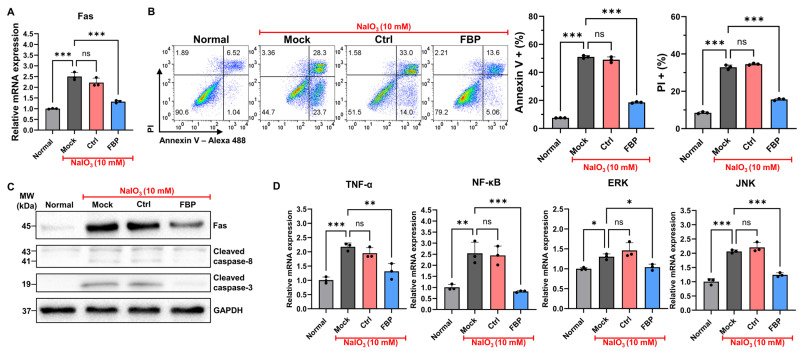
FBP reduces Fas-mediated apoptosis and inflammation. (**A**) Relative expression of *Fas* assessed using RT-PCR following NaIO_3_ treatment. (**B**) Flow cytometry analysis of ARPE-19 cells using annexin V staining. Cells were treated with nonspecific peptide (Ctrl) or FBP. Dot plot (**left**) and a bar graph (**right**) illustrate annexin V- and PI-stained cells. (**C**) Expression of apoptosis-related proteins was assessed using Western blot analysis. (**D**) Relative expression of cytokine and signaling pathway-related genes assessed using RT-PCR. Data are presented as mean ± SD (*N* = 3 per group). * *p* < 0.05, ** *p* < 0.01, *** *p* < 0.001; ns, not significant.

To validate the cell-protective effects of FBP under conditions mimicking dry AMD, we treated ARPE-19 cells with NaIO_3_. In NaIO_3_-treated mock and nonspecific peptide-treated control cells, the expression of Fas was increased, whereas it was reduced in FBP-treated cells (Figure 1A). Following the confirmation of Fas upregulation in NaIO_3_-treated ARPE-19 cells, we assessed the efficacy of FBP in inhibiting apoptosis. NaIO_3_-treated cells exhibited increased apoptosis, whereas FBP-treated cells exhibited a statistically significant decrease in apoptosis compared with that in the mock group (Figure 1B). The control peptide exhibited no protective effects against apoptosis, similar to the mock group. Intriguingly, FBP inhibited both early- and late-stage apoptosis, as evidenced by annexin V and PI staining, respectively. Further investigation into the levels of apoptotic proteins in ARPE-19 cells treated with control peptide or FBP revealed upregulation of cleaved caspase-3 and 8 proteins in the mock and control peptide-treated cells. In contrast, the FBP-treated cells exhibited a significant decrease in the levels of cleaved caspase-3 and 8, which are involved in both apoptotic (caspase-8) and necrotic (caspase-3) pathways, compared with that in the mock and control peptide-treated cells (Figure 1C). Additionally, NaIO_3_-treated ARPE-19 cells showed induction of inflammatory cytokines such as TNF-α and increased expression of transcription factors NF-κB, ERK, and JNK, which are associated with inflammation and the apoptosis pathway in the retina. Conversely, FBP-treated retinal cells exhibited decreased levels of NF-κB, ERK, and JNK (Figure 1D).

### 3.2. Evaluation of FBP Delivery via Eye Drops in a Murine Model of NaIO_3_-Induced Retinal Degeneration

**Figure 2:**
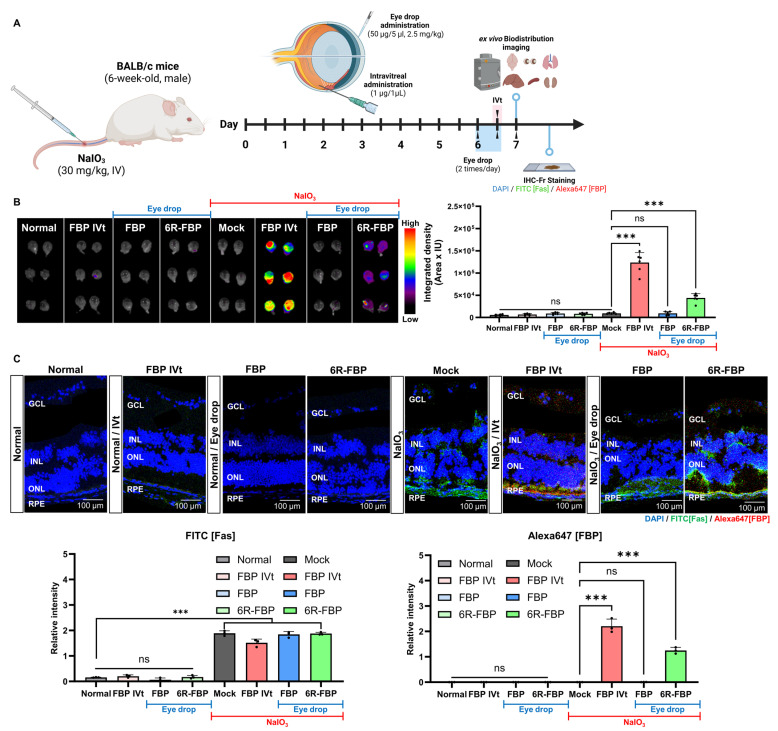
Eye drop formulation of 6R-FBP for retinal delivery in a NaIO_3_-induced retinal degeneration mouse model. (**A**) A scheme of experiments outlining the assessment of the efficiency of 6R-FBP delivery in a NaIO_3_-induced retinal degeneration mouse model. (**B**) Representative fluorescence image of fluorescence-labeled FBP in the left and right eyes (*n* = 3 per group). Cumulative data for estimating fluorescence signals from the eye based on three independent experiments (*n* = 3 per group). (**C**) Confocal microscopy images showing Fas (FITC-stained) and FBP (Alexa647-stained) delivery in normal and NaIO_3_-induced retinal degeneration mice; nuclei were stained with DAPI. FBP binding was assessed using immunohistochemistry. Data are presented as mean ± SD (*N* = 3 per group). *** *p* < 0.001; ns, not significant.

Following confirmation of the therapeutic effect of FBP in oxidative-stress-induced ARPE-19 cells, we investigated the biodistribution of FBP delivered through eye drops using Alexa647-tagged FBP (Figure 2A). No fluorescence was observed in the eyeballs of NaIO_3_-treated and normal mice upon the administration of Alexa647-tagged FBP via eye drops (Figure 2B). In contrast, in the NaIO_3_-treated mice group, intravitreal injection of FBP resulted in successful FBP delivery to the retina, which was evidenced by strong fluorescence signals in the posterior part of the eye (Figure 2B). To enhance the retinal delivery of the FBP peptide without intravitreal administration, we harnessed CPP, previously suggested for ocular peptide delivery [51,57,58,59]. Cytotoxicity testing in retinal cells revealed lower toxicity for FBP conjugated with six or fewer arginine units (6R-FBP) compared with that of the nona-arginine conjugate (9R) (Figure S3). The 6R-FBP administered through eye drops localized to the Fas-expressing posterior part of the mouse eye, exhibiting strong fluorescence signals in NaIO_3_-induced retinal degeneration mice but not in normal mice (Figure 2B). Moreover, no difference in fluorescence signal was observed in other organs in all the groups compared with that in the normal group, confirming that FBP was not transferred to any other organ but the eyes. Only the FBP intravitreal and 6R-FBP eye drop groups demonstrated successful delivery to the eyes (Figure 2B). Consistent with the intravitreal application of FBP, the eye drop application successfully delivered 6R-FBP to the eye in Fas-expressing NaIO_3_-induced retinal degeneration mice. To further confirm the delivery of FBP, we employed confocal imaging. Almost no fluorescence signal was noted in mice administered FBP through eye drops without CPP. In contrast, intravitreally injected mice and those receiving eye drops with 6R-FBP displayed strong fluorescence signals, indicating successful delivery only in NaIO_3_-induced retinal degeneration mice; no fluorescence signal was detected in normal mice (Figure 2C).

### 3.3. Therapeutic Efficacy of 6R-FBP Eye Drops in Attenuating Retinal Degeneration in a NaIO_3_-Induced Retinal Degeneration Mouse Model

**Figure 3:**
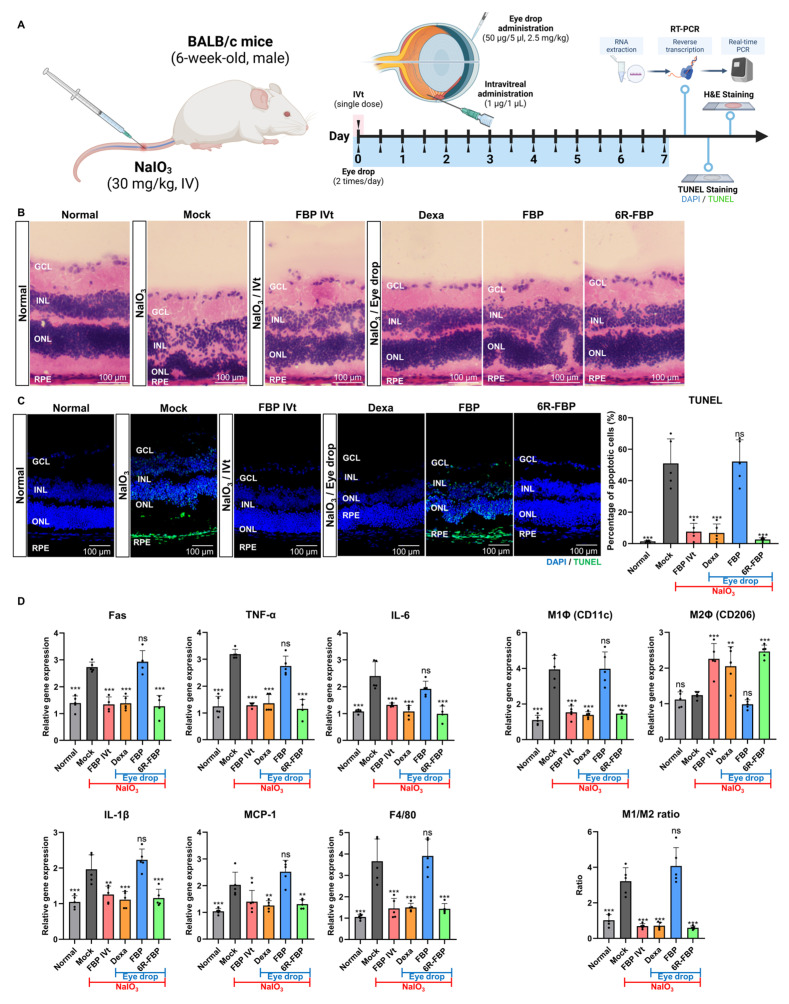
6R-FBP eye drops attenuate retinal degeneration in a NaIO_3_-induced retinal degeneration mouse model. (**A**) A scheme of experiments outlining the assessment of the therapeutic efficiency of 6R-FBP in a NaIO_3_-induced retinal degeneration mouse model. (**B**) Assessment of the therapeutic effect in histological analysis using H&E staining. (**C**) Assessment of the therapeutic effect on apoptosis inhibition using TUNEL staining. (**D**) Assessment of the therapeutic effect on cytokine levels assessed using qPCR. Data presented as mean ± SD (*N* = 3 per group). The data in (**C**,**D**) were analyzed using one-way ANOVA versus mock group * *p* < 0.05, ** *p* < 0.01, *** *p* < 0.001; ns, not significant.

Following the successful delivery of 6R-FBP through eye drops, its efficacy was evaluated in NaIO_3_-induced retinal degeneration mice (Figure 3A). The mock group exhibited a reduction and distortion in the RPE layer of the retina compared with that in the normal group. Eye drop application of the control peptide and FBP had no therapeutic effect, whereas eye drop application of dexamethasone (Dexa) and 6R-FBP, as well as intravitreal injection of FBP, exhibited therapeutic effects, resulting in the maintenance of the integrity of the retinal layer, as evidenced in the H&E staining of sections (Figure 3B). For apoptosis analysis, TUNEL staining of cryosections was performed, wherein cell nuclei were stained in blue (DAPI), and dead cells were stained in green (FITC). The mock group exhibited a higher apoptosis level, increased retinal collapse, and a highly distorted retinal layer in both H&E staining and the TUNEL assay compared with that in the normal group. Eye drop application of the control peptide and FBP had no therapeutic effect, as evidenced from retinal layer distortion and higher apoptosis levels. Conversely, the Dexa-, 6R-FBP-eye-drop-, and FBP-intravitreal-treated groups showed maintenance of the retinal layer and lower apoptosis levels (Figure 3C). The NaIO_3_-induced retinal degeneration mock group showed increased expression of Fas, inflammatory cytokines (TNF-α, IL-6, and IL-1β), MCP-1, and macrophages (F4/80), and an elevated M1 (CD11c)/M2 (CD206) macrophage ratio compared with that in the normal mouse group (Figure 3D). However, eye drop application of Dexa and 6R-FBP and intravitreal injection of the FBP resulted in decreased expression of Fas receptor, inflammatory cytokines, and macrophages and increased levels of M2 macrophages compared to the mock group (Figure 3D).

### 3.4. FBP Attenuated Retinal Degeneration in the NaIO_3_-Induced Retinal Degeneration Rabbit Model

**Figure 4:**
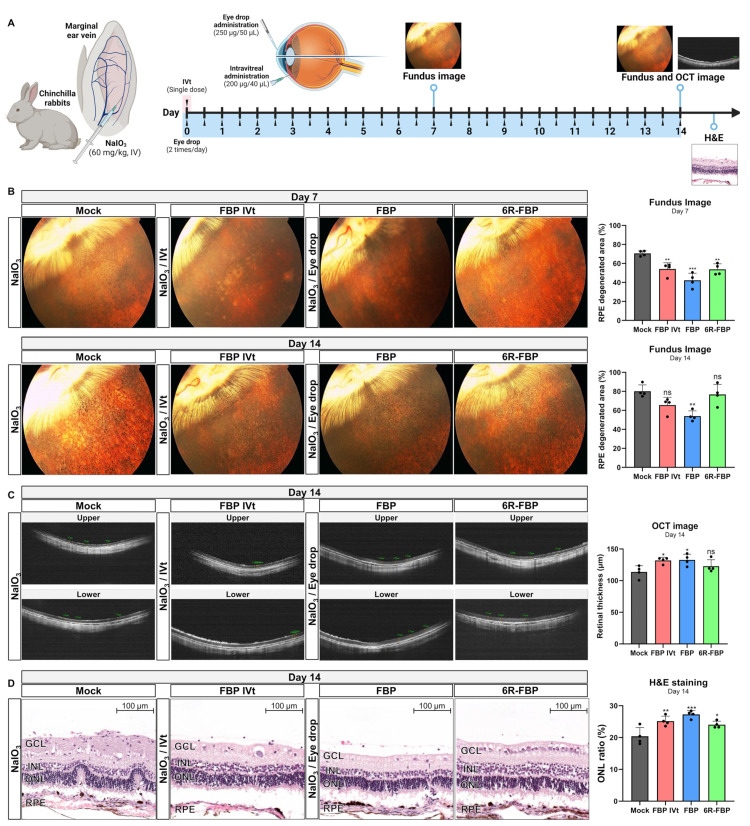
FBP attenuates retinal degeneration in a NaIO_3_-induced retinal degeneration rabbit model. (**A**) A scheme of experiment outlining the assessment of the therapeutic efficiency of FBP in a NaIO_3_-induced retinal degeneration rabbit model. (**B**) Assessment of the therapeutic effect in the RPE-degenerated area using fundus imaging. (**C**) Assessment of the therapeutic effect in retinal thickness analysis using OCT. (**D**) Assessment of the therapeutic effect using ONL ratio analysis through H&E staining. Data are presented as mean ± SD (*N* = 4 per group). * *p* < 0.05, ** *p* < 0.01, *** *p* < 0.001; ns, not significant.

To further confirm the therapeutic effects of FBP in another animal model, we conducted experiments, which were outsourced to KNOTUS, on a rabbit NaIO_3_-induced retinal degeneration model (Figure 4A). Male rabbits were intravenously administered 60 mg/kg of NaIO_3_ on Day 1 to induce dry AMD. Subsequently, FBP was applied intravitreally to one eye once during the 14-day experimental period or twice a day for 14 days using an eye drop. Additionally, 6R-FBP was applied as an eye drop twice a day for 14 days, as illustrated in Figure 4A. At both 7 and 14 days following the induction of NaIO_3_-induced retinal degeneration, we assessed the extent of degeneration of the RPE layer via fundus imaging (Figure 4B). The intravitreal injection of FBP, FBP, and 6R-FBP eye drops caused a significant reduction in RPE degeneration compared with that in the mock treatment (Figure 4B). Rabbits treated with FBP exhibited the most promising inhibition of RPE degeneration. The representative fundus images (left) and cumulative data (right) for days 7 and 14 indicated the prevention of RPE degeneration by intravitreal injection of FBP and by the FBP and 6R-FBP eye drops. On day 14 after NaIO_3_-induced retinal degeneration induction, the level of RPE degeneration remained significantly lower in rabbits intravitreally injected with FBP and in those treated with eye drops compared with that in the mock group. On the contrary, in rabbits treated with 6R-FBP, the levels of RPE degeneration were similar to those in the mock group. In these rabbit experiments, the application of FBP eye drops significantly delayed RPE degeneration on day 14, showing better therapeutic effects than the intravitreal FBP treatment. In contrast with the negative effects observed in the FBP eye drop treatment of the rodent NaIO_3_-induced retinal degeneration model, in the rabbit model, this treatment protected against RPE degeneration (Figure 4B). Furthermore, on the 14th day after the induction of NaIO_3_-induced retinal degeneration, the thickness of the retina measured using OCT imaging was reduced in both the FBP eye drop group and intravitreal FBP group compared with that in the mock group (Figure 4C). Both fundus and OCT imaging confirmed the therapeutic effects of the FBP eye drop and intravitreal administration of FBP, indicating that FBP inhibits RPE degeneration when delivered to the retinal area.

Next, the right eyes of the rabbits were harvested, and specimens were prepared for H&E staining. The proportion (%) of the thickness of the ONL to the total thickness of the retina was calculated using an optical microscope (Figure 4D). Consistent with previous data, the delivery of FBP via intravitreal injection or FBP eye drops, as well as 6R-FBP eye drops, was associated with the maintenance of the ONL in the retinal area, indicative of inhibition of NaIO_3_-induced retinal degeneration compared with that in the mock group. Based on these in vivo results, we confirmed that FBP ameliorates NaIO_3_-induced retinal degeneration in the rabbit model and exhibits a therapeutic effect.

### 3.5. Impact of Peptide Size on the Efficacy of Eye Drop in the NaIO_3_-Induced Retinal Degeneration Mouse Model

**Figure 5:**
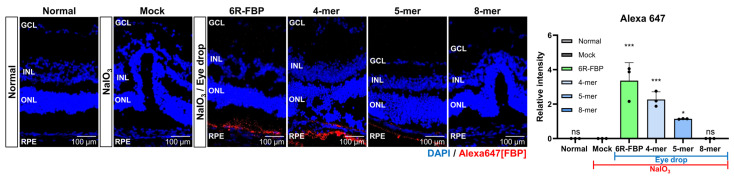
Efficient retinal delivery of FBP eye drop in an NaIO_3_-induced retinal degeneration mouse model. Size-dependent delivery of FBP, demonstrating the capability of the small-sized FBP eye drop to reach the retina. 4-mer, 5-mer truncated FBP, 8-mer FBP, and 6R-FBP labeled with fluorescent dye were applied as eye drops to NaIO_3_-induced retinal degeneration mice. Confocal microscopy images showing FBP (Alexa647-stained) delivery in NaIO_3_-induced retinal degeneration mice; nuclei were stained with DAPI. Data are presented as mean ± SD (*N* = 3 per group). * *p* < 0.05, *** *p <* 0.001; ns, not significant.

Our investigations into the therapeutic effects of FBP when applied as an eye drop in NaIO_3_-induced retinal degeneration rabbits compared with those in NaIO_3_-induced retinal degeneration mice revealed notable differences. The observed variations may be attributed to differences in eye structure among species, including variances in the blood–retinal barrier (BRB) [60]. Additionally, the significantly shorter half-life of the vitreous humor in mice—approximately 10 times shorter than that in rabbits—is believed to contribute to these disparities [61]. Recognizing the variations in eye structures across different animal species, we hypothesized that the efficiency of peptide delivery may be influenced by the size of the peptide. To explore whether peptide size plays a role in determining the efficacy of eye drop application in the NaIO_3_-induced retinal degeneration model, we utilized modified FBP peptides of varying lengths (Table 1). These modifications included 4-mer and 5-mer FBP, which demonstrated binding to Fas-expressing Jurkat cells (Figure S4A) but could not inhibit Fas/FasL-mediated apoptosis, unlike the 8-mer FBP peptide (Figure S4B). To investigate whether peptide size posed a challenge in delivering the 8-mer FBP peptide in the rodent model, we administered Alexa647-tagged 4-mer and 5-mer FBP as eye drops to NaIO_3_-induced retinal degeneration mice. Contrary to previous data showing that the 8-mer FBP was not delivered without CPP (Figure 2B,C), we observed that the 4-mer FBP was successfully delivered, similar to that in the 6R-FBP group, as evidenced from the robust fluorescence signals (Figure 5 and Figure S4A). These findings suggest that intraocular delivery of peptide drugs may vary depending on the size of the peptide, highlighting the importance of considering peptide size in the development of effective eye drop applications for AMD treatment.

## 4. Discussion

In this study, we investigated the therapeutic potential of FBP in the context of oxidative stress-induced damage by mimicking the conditions of dry AMD. The motivation for this exploration stemmed from our earlier findings, demonstrating the effective inhibition of Fas/FasL ligation by FBP in neuronal cell death caused by ischemic stroke [54]. Through a comprehensive exploration of the effects of FBP on retinal cells in AMD, its intraocular delivery, and considerations regarding peptide size, we obtained promising insights relevant for potential applications in the treatment of retinal diseases. We initially established the safety and efficacy of FBP, which inhibited both early and late-stage apoptosis in NaIO_3_-treated ARPE-19 cells. This not only highlights the potential of FBP in ameliorating cellular damage but also underscores its role in reducing inflammation associated with dry AMD. Despite oxidative stress being the major driver of AMD progression [43], our findings indicate that blocking Fas/FasL signaling with FBP could impede AMD progression by targeting both Fas-mediated apoptosis and ROS-mediated inflammatory death pathways (Figure A1). While apoptosis is the primary outcome of the Fas/FasL interaction, this pathway can also contribute to inflammation through JNK- and ERK-induced TNF-α [62,63]. When cells undergo apoptosis via Fas/FasL signaling, they activate various proteins, such as ASK-1, ERK, p53, and NF-κB, which trigger the intrinsic apoptosis pathway. Initial cell damage activates p53 and NF-κB, resulting in the upregulation of Fas. As a result of Fas signaling, the Fas receptor is overexpressed, and the inhibition of Fas signaling by FBP leads to a decrease in Fas receptor expression. The engagement of Fas to FBP prevents the interaction of Fas to FasL, thereby reducing Fas surface signaling as the Fas molecule that is not engaged may become internalized or degraded [20,23,64]. Fas activates NF-κB, resulting in the expression of the initial stages of ROS signaling by cytokines. Thus, the approach employed by us results in the blocking of the Fas/FasL interaction, resulting in the inhibition of inflammation and apoptosis.

Intraocular delivery poses a substantial challenge in ocular therapeutics [48]. We explored FBP delivery via eye drops and noted successful intravitreal delivery but limited efficacy with conventional eye drops. To overcome this, we harnessed hexa-arginine as a useful moiety for the delivery of FBP. The modified 6R-FBP exhibited successful localization in the Fas-expressing posterior part of the eye in NaIO_3_-induced retinal degeneration mice. This strategy not only highlights the importance of considering delivery mechanisms but also offers a potential solution for targeted ocular drug delivery, as used by others [51]. However, we chose hexa-arginine to minimize the number of positively charged arginine residues, thus avoiding cell toxicity and maximizing the efficiency of CPP (Figure S3). The 6R-FBP eye drops are efficiently localized in the Fas-expressing posterior part of the mice’s eyes in the RPE area, with strong fluorescence signals in NaIO_3_-induced retinal degeneration mice but not in normal mice, without causing cell death. Administration of 6R-FBP eye drops to NaIO_3_-induced retinal degeneration mice resulted in a significant reduction in retinal degeneration compared with that in the control group, which exhibited retinal collapse, the presence of inflammatory cytokines, and an increase in M1 macrophages (Figure 3). However, characterizing infiltrating cells based solely on CD11c/CD206 qPCR expression may present inadequacies. Therefore, there is a need for further characterization at the protein and cellular levels using additional surface marker proteins. Our results strongly suggest that 6R-FBP effectively blocks the Fas/FasL interaction, leading to the inhibition of apoptosis and inflammation, thereby positioning 6R-FBP as a promising inhibitor of AMD progression. These findings align with previous research demonstrating that Fas knockout mice are resistant to AMD progression, highlighting the potential benefits of blocking Fas activity in the retina for AMD treatment [31]. While pharmacologic inhibition of the Fas/FasL interaction has been achieved through intraocular injection of macromolecules, such as anti-Fas antibodies, soluble FasL, and Fas receptor antagonists, their application is limited by severe side effects [65]. In contrast, the application of small peptide mimetics that block the Fas–FasL interacting complex through eye drops shows promise for clinical application [66,67].

We further confirmed the therapeutic effects of FBP in a rabbit NaIO_3_-induced retinal degeneration model. FBP attenuated retinal degeneration in NaIO_3_-induced retinal degeneration rabbits. Both 6R-FBP and the FBP eye drops efficiently inhibited NaIO_3_-induced retinal degeneration progression. Without CPP, FBP itself distinctly inhibited retinal degeneration, as evidenced from the ratio of the thickness of the ONL to the total thickness of the retina, H&E staining, and optical coherence tomography imaging of RPE. To investigate the observed variation in the delivery of the FBP peptide, which was ineffective in the rodent retina but successful in the rabbit NaIO_3_-induced retinal degeneration model, we made modifications to the FBP peptide. The modified peptide was administered as an eye drop to NaIO_3_-induced retinal degeneration mice for further assessment. An intriguing observation emerged when exploring the impact of peptide size on intraocular delivery in the two different NaIO_3_-induced retinal degeneration models. The successful delivery of 4-mer FBP via eye drops, in contrast to the challenges encountered in the delivery of the 8-mer FBP without CPP, suggests a size-dependent variability in intraocular delivery (Figure 5). This finding supports the notion that interspecies differences in ocular delivery could contribute to the insufficient efficacy of eye drop drugs observed in clinical studies [57]. The variations in FBP delivery and effects across species were interpreted by considering differences in the structure of the eye and blood vessels [57,60]. Additionally, mice have a tenfold shorter half-life of the vitreous humor compared to rabbits [61]. Therefore, further research is needed to precisely determine whether these differences are influenced solely by the size of the peptide or by other factors, such as concentration and flow rate [61]. Therefore, ongoing efforts in some studies focus on increasing the vitreous half-lives of drugs [68,69], and subsequent research is needed for peptide modifications to enhance the effects of FBP. Additionally, it is possible that another R-length FBP may be more suitable for rabbits; so, further research is needed to investigate the differences in efficacy of FBP based on R-length derivatives.

We found that the degeneration of the retina in our animal model could be treated through the delivery of FBP. This discovery suggests the potential application of FBP in the treatment of AMD. Moreover, the disparities noted in FBP delivery and its effects across different species can be attributed to variations in eye and blood vessel structures. In the experiment involving a truncated version of FBP, we discovered that the size of the peptide can account for the difference in ocular delivery among animal species. Nonetheless, additional research is needed to ascertain whether this influence is specifically due to size or other factors, such as concentration or flow rate [57,61]. Our experimental findings provide valuable insights for the future advancement of macular degeneration treatments. The observed differences in the efficiency of intraocular delivery of peptides across species, which are linked to the peptide size, warrant additional exploration. If an eye drop formulation of FBP is effective in a primate model, it may pave the way for novel developments in peptide eye drops applicable for use in humans [70].

In summary, our study delves into the multifaceted aspects of FBP, encompassing its cell-protective effects, innovative intraocular delivery strategies, and considerations of peptide size. These findings provide valuable insights that should be relevant to the ongoing efforts to develop effective treatments for retinal diseases, particularly AMD. Further investigations into the underlying mechanisms and clinical translatability of these findings will be crucial for advancing the field of ocular therapeutics.

## 5. Conclusions

This study offers a comprehensive exploration into the therapeutic potential of the Fas-blocking peptide (FBP) for dry age-related macular degeneration (AMD). In a series of experiments, the inhibition of Fas-mediated signaling was found to be associated with a significant reduction in retinal cell death, suggesting a promising therapeutic approach for dry AMD. The intravitreal injection of FBP effectively mitigated retinal pigment epithelium (RPE) degeneration in an NaIO_3_-induced retinal degeneration rodent model. Furthermore, the eye drop formulation of 6R-FBP exhibited efficient delivery to the RPE, effectively inhibiting NaIO_3_-induced retinal degeneration in the rodent model. Notably, the naked form of FBP could reach the RPE and prevent degeneration in the NaIO_3_-induced retinal degeneration rabbit model. The exploration of size-dependent ocular delivery through an eye drop formula should be considered across several animal models. The application of an antiapoptotic peptide via eye drop introduces novel prospects for therapeutic interventions in human dry AMD.

## Figures and Tables

**Table 1:** Sequences of the synthesized peptides.

Peptide Name	Peptide Sequence	Molecular Weight (Da)
FBP	YCDEHFCY	1079.1
4-mer	DEHF	546.5
5-mer	CDEHF	649.7
4R-FBP	RRRRGGGGYCDEHFCY	1932.1
5R-FBP	RRRRRGGGGYCDEHFCY	2088.3
6R-FBP	RRRRRRGGGGYCDEHFCY	2244.5
9R-FBP	RRRRRRRRRGGGGYCDEHFCY	2713.1
Ctrl	GGGRRGGG	672.7

**Table 2:** Sequences of primer pairs used for real-time PCR.

Target Gene	Primer Sequence
*Fas(CD95)*	Forward: 5′-GGAGGTGGTGATAGCCGGTAT-3′
	Reverse: 5′-TGGGTAATCCATAGAGCCCAG-3′
*TNF-α*	Forward: 5′-GGTGCCTATGTCTCAGCCTCTT-3′
	Reverse: 5′-GCCATAGAACTGATGAGAGGGAG-3′
*IL-6*	Forward: 5′-TACCACTTCACAAGTCGGAGGC-3′
	Reverse: 5′-CTGCAAGTGCATCATCGTTGTTC-3′
*CD11c*	Forward: 5′-TTCTTCTGCTGTTGGGGTTTG-3′
	Reverse: 5′-CAACCACCACCCAGGAACTAT-3′
*CD206*	Forward: 5′-GGCAGGATCTTGGCAACCTAGTA-3′
	Reverse: 5′-CCTTTCTTCCGACTCTTCACCC-3′
*IL-1β*	Forward: 5′-TTCAGGCAGGCAGTATCACTC-3′
	Reverse: 5′-GAAGGTCCACGGGAAAGACAC-3′
*MCP-1*	Forward: 5′-TAAAAACCTGGATCGGAACCAAA-3′
	Reverse: 5′-GCATTAGCTTCAGATTTACGGGT-3′
*F4/80*	Forward: 5′-CTGAGGATGAATTCCCGTGT-3′
	Reverse: 5′-GTCTCGGATGCTTCCACAAT-3′
*GAPDH*	Forward: 5′-AGGTCGGTGTGAACGGATTTG-3′
	Reverse: 5′-TGTAGACCATGTAGTTGAGGTCA-3′

## Data Availability

The original contributions presented in the study are included in the article/Supplementary Materials; further inquiries can be directed to the corresponding authors.

## Supplementary Materials

The following supporting information can be downloaded at: https://www.mdpi.com/article/10.3390/cells13060548/s1, Figure S1: Evaluation of peptide toxicity in ARPE-19 cells using the CCK-8 assay; Figure S2: Concentration-dependent cell viability in NaIO_3_-induced ARPE-19 cells; Figure S3: Evaluation of the toxicity of cell-penetrating peptide (CPP) in ARPE-19 cells using the CCK-8 assay; Figure S4: Analysis of size-dependent FBP binding in Jurkat cells.
