# Supplementary material for: Eye Drop with Fas-Blocking Peptide Attenuates Age-Related Macular Degeneration
Source: Cells. 2024 Mar 20;13(6):548. doi: 10.3390/cells13060548 (PMC10969560; doi:10.3390/cells13060548)
Supplement: Supplementary file 1 [file cells-13-00548-s001.zip › cells-2860631-supplementary.pdf]

Supplementary Materials

Eye Drop with Fas-Blocking Peptide Attenuates Age-Related Macular Degeneration

Yujong Yi [1,†], Seon-Hong Pyun [1,†], Chae-Yeon Kim [1], Gyeongju Yun [1], Eunhwa Kang [1],Seoyoun Heo [1], Irfan Ullah [2,*] and Sang-Kyung Lee [1,*]

Total Supplementary Figures (S1~S4)

Figure S1. Peptide toxicity test by CCK-8 assay in ARPE-19 cells.

Figure S2. Concentration-dependent cell viability in NaIO3-induced ARPE-19 cells.

Figure S3. Cell-Penetrating Peptide (CPP) toxicity test by CCK-8 assay in ARPE-19 cells.

Figure S4. Size-dependent FBP binding test in Jurkat cells.

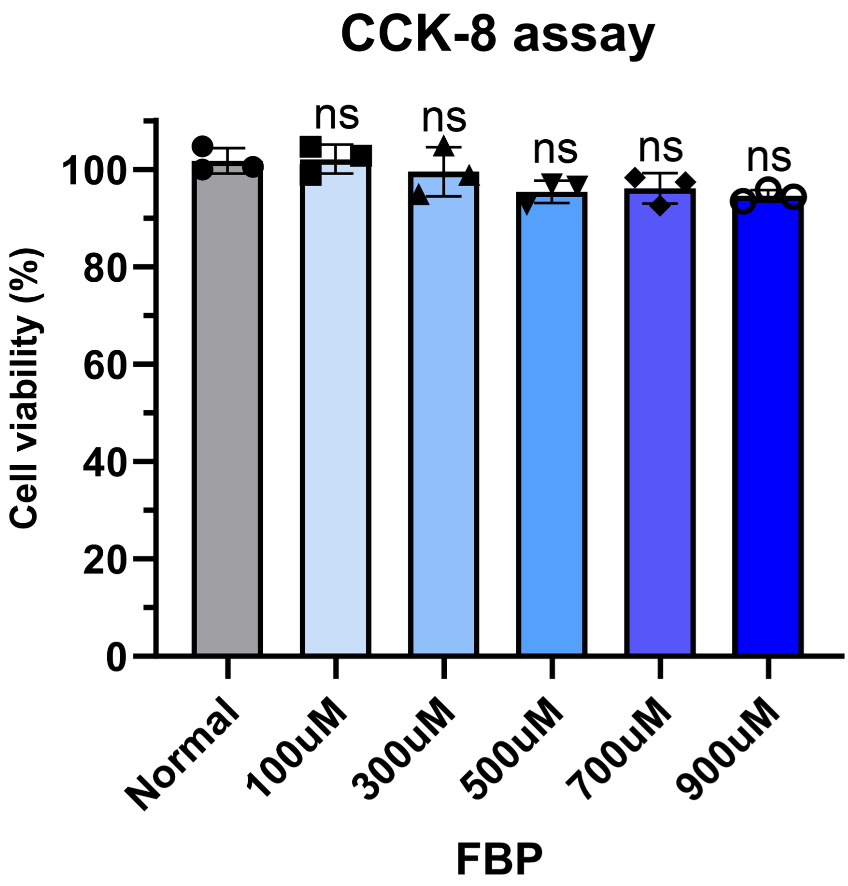

Figure S1. Peptide toxicity test by CCK-8 assay in ARPE-19 cells. Peptide toxicity was assessed using CCK-8 assay in ARPE-19 cell. The data are presented as the mean ± SD (N=3 per group). ns indicates not significant.

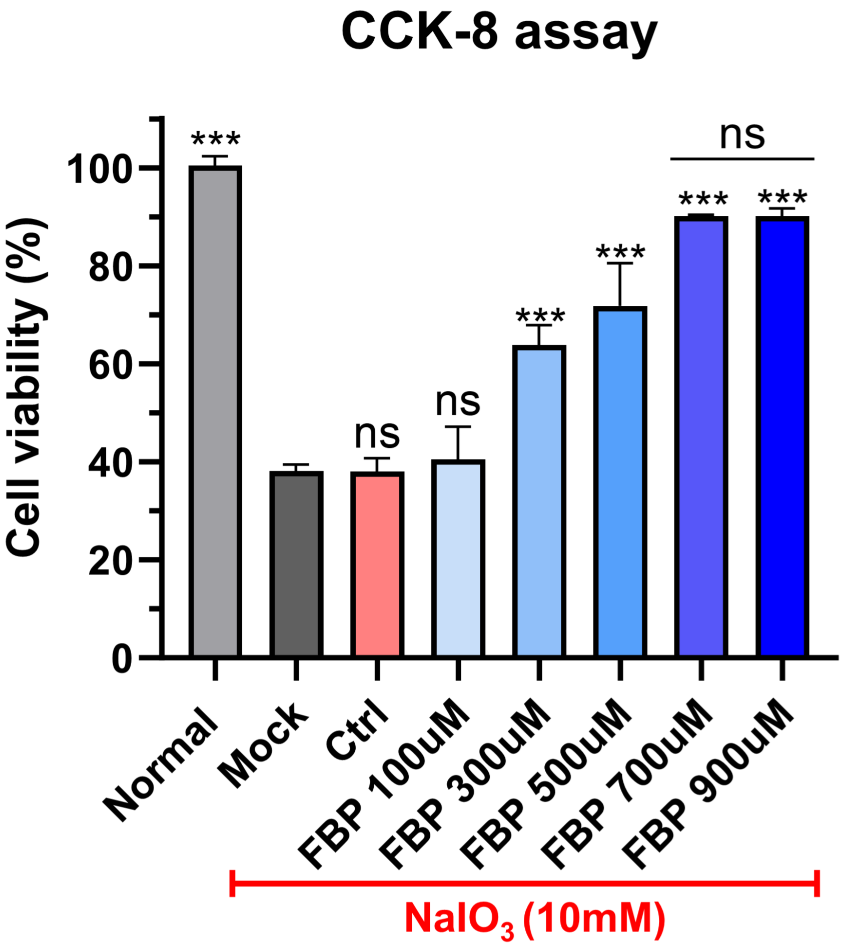

Figure S2. Concentration-dependent cell viability in NaIO3-induced ARPE-19 cells. Cell viability under concentration-dependent conditions was evaluated in NaIO3-induced ARPE-19 cells. The data are presented as the mean ± SD from three independent experiments. ***P<0.001, and ns indicates not significant.

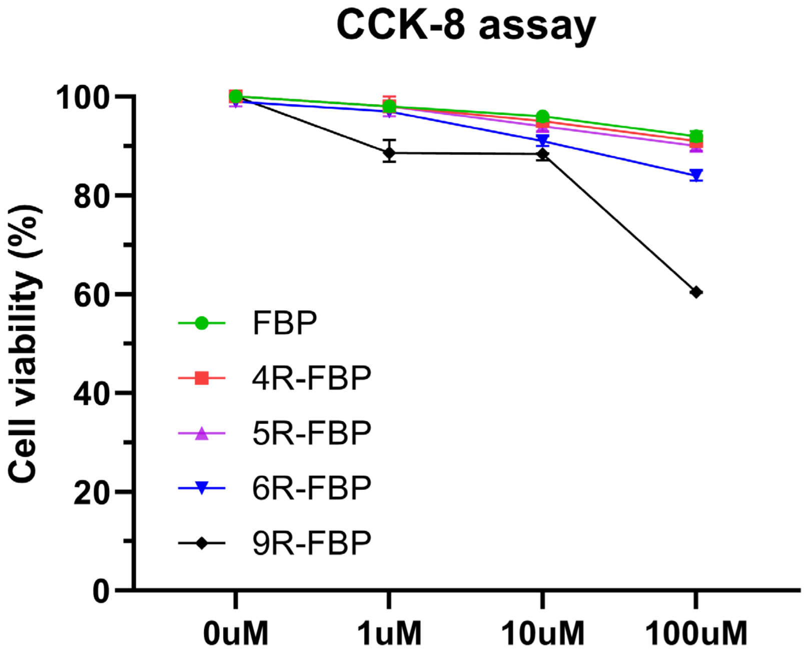

Figure S3. Cell-penetrating peptide (CPP) toxicity test by CCK-8 assay in ARPE-19 cell. CPP toxicity was assessed using the CCK-8 assay in ARPE-19 cell. The data are presented as the mean ± SD (N=3 per group).

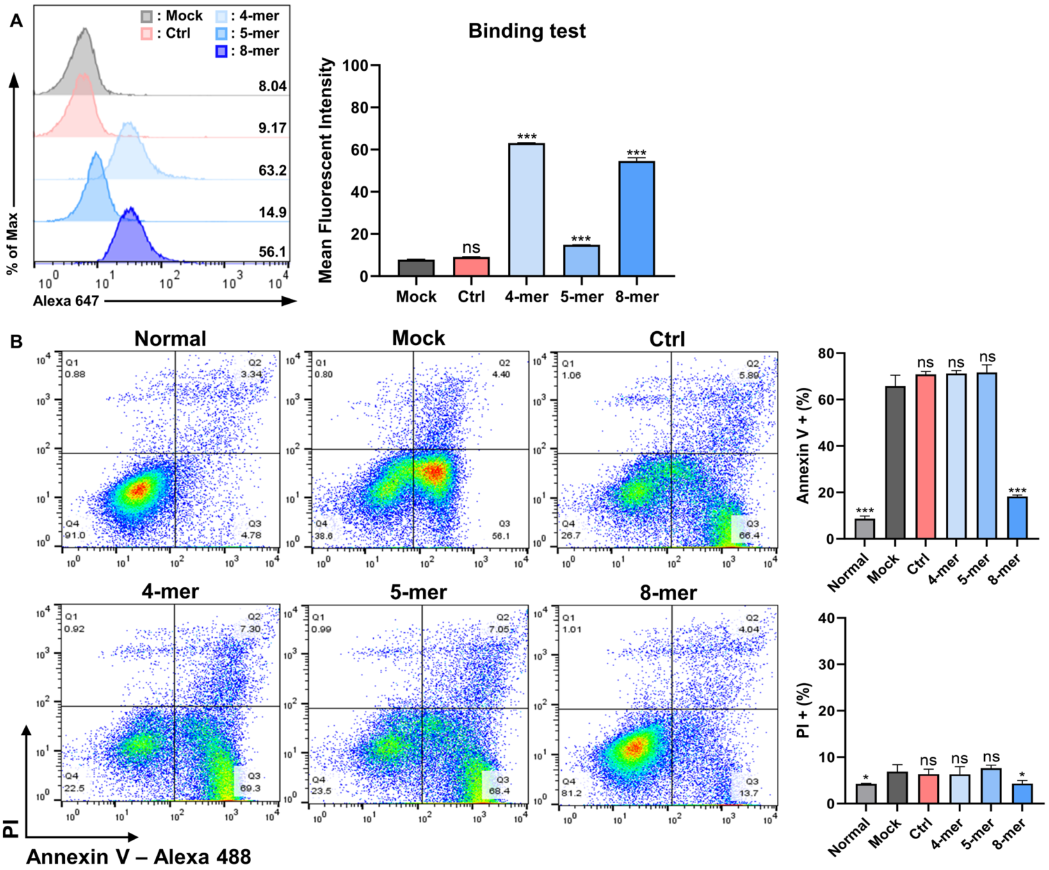

Figure S4. Size-dependent FBP binding test in Jurkat cells. (A) Size-dependent FBP flow cytometry analysis of binding in Jurkat cells. (B) Size-dependent FBP flow cytometry analysis of apoptosis in Jurkat cells. The data are presented as the mean ± SD (N=3 per group). *P<0.05, ***P<0.001, and ns indicates not significant.
